# Ecosphere Management Model of Farmer Cooperatives Based on Intelligent Data Sampling Technology

**DOI:** 10.1155/2022/1549275

**Published:** 2022-03-24

**Authors:** Ting Fang, Xiaoming Liao, Ming Fang

**Affiliations:** ^1^School of Public Administration, Nanchang University, Nanchang 330031, China; ^2^School of Marxism, Nanchang University, Nanchang 330031, China

## Abstract

In order to improve the operation effect of farmer cooperatives, this paper combines the intelligent data sampling technology to analyze the ecological circle operation mode of farmer cooperatives. Moreover, this paper strives to promote the ecosphere business model, accelerate regional development, build agricultural pastoral complex projects and in-depth study TI-ADC modeling, error estimation, mismatch compensation, and other technologies, and carry out engineering realization. In addition, this paper uses technology to analyze intelligent data and builds a system structure based on the actual needs of the farmer cooperative ecosphere management. Finally, this paper analyzes the structure and flow of the data processing layer. The test results show that the ecosystem business model of farmer cooperatives based on intelligent data sampling technology proposed in this paper has good results.

## 1. Introduction

Farmers' professional cooperatives (hereinafter referred to as “cooperatives”) are rooted in China's rural society and are a low-cost form of organization that improves the degree of organization of Chinese farmers and promotes farmers' poverty alleviation and income. Under the dual effects of farmers' demand and the government's efforts to promote [1], while the number of cooperatives is increasing rapidly, they also face many doubts, such as qualitative drift, separation of name and reality, restraining economies of scale and increasing competitiveness, and hindering the connection between small farmers and modern agricultural development [2]. It can achieve element complementation and organizational coordination and reduce the degree of heterogeneity of cooperatives, which is another way to break the anomie development of cooperatives. Moreover, it is also an important breakthrough for cooperatives to overcome resource constraints and scale traps [3] and has received attention and attention from both political and academic circles.

From the perspective of organizational ecology, the establishment of an industrial organization is a process of competing for scarce public resources under the influence of factors such as the ecology, imprint, and niche capacity of the founders to form a mutually beneficial symbiotic relationship between industrial organizations. Organizational evolution is often hindered by organizational inertia and resource specificity. There is a balance between the breadth of the organization's niche and the organization's adaptability to the environment, and population density will produce two opposing processes for the organization: legitimacy and competitiveness. Competition is an important factor that affects the death of an organization, but not all competition leads to death. The relationship density representing the formal relationship between members of the population and the number of key roles will reduce the death rate of the organization. Management continuity will increase organizational mortality, but as the organization ages, the impact of management continuity on organizational mortality will gradually decrease. It can be seen that organizational ecology believes that the process of organizational growth and evolution is a process in which initial conditions are used as prefactors to produce a lasting impact when the organization is established. In the “natural selection” process of the environment to the organization, the growth and evolution of the organization is the change of the organization in the environment from one state to another. In terms of how the initial conditions exert their influence, how their generation mechanism unfolds, and the identification and coupling of organizational endogenous variables and exogenous environmental variables, we find that there is room for further research. At present, organizational ecology is widely used in corporate research and rarely used in cooperative research.

On the one hand, farmer cooperatives are similar to corporate organizations, pursuing reorganized potential profits, and forming a mutually beneficial symbiosis with other entities in the agricultural supply chain, such as agricultural material suppliers, agricultural product processors, logistics companies, local governments, and other industrial organizations. In the establishment and evolution of ecosystems, farmers' cooperatives seek both competitiveness and legitimacy, and the initial conditions and environment embedded in the establishment of farmers' cooperatives shape the organizational imprint of farmers' cooperatives and promote farmers' cooperatives. Select the appropriate niche in the process of adapting and matching with the environment. On the other hand, farmers' cooperatives are different from investor-owned enterprises but are recooperative economic organizations with the same owner and patrons. The development of farmers' cooperatives is often deeply influenced by institutional actors, the main body of the agricultural supply chain, and internal member societies. However, loose or tight internal ecological circles will be formed between farmers' cooperatives and their member societies, as well as between their internal member societies, and the internal ecological circle and ecological niche will be coupled or separated, thus forming different evolutionary paths of farmers' cooperatives.

This paper combines the intelligent data sampling technology to analyze the business model of the farmer cooperative ecosystem and builds an intelligent system to analyze the further development of the farmer cooperative's ecological business model.

## 2. Related Work

From the visual analysis of cooperative research hotspots in the past 20 years, it can be seen that the research on cooperatives in China is still at the level of epistemic cognition, and the problems related to cooperative research can be classified into four categories. One is the research on the rationality of the existence of cooperatives. Scholars have studied the rationality of cooperative development in depth from the perspectives of industrial development, farmers' rights and interests, and institutional arrangements, drawing on the theories of transaction costs, property rights, and principal-agent in Western economics [4]. In the view of scholars, cooperatives are the most effective agricultural institutional arrangement that combines the family management system and the cooperative system in agriculture. They can not only reduce transaction costs such as information, supervision, and execution through economies of scale, but also make up for the shortcomings of market mechanisms. As well as supplementing the functions of government departments [5], cooperatives can effectively guide farmers to connect with the market, which not only provides organizational guarantees for rural economic development, but also is the result of rational choices for “rational small farmers” to cope with production risks [6]. The second is the research on the nature of cooperatives. There has been a long-standing debate on whether cooperatives are enterprises or mutual aid and self-benefiting organizations of farmers. Scholars have discussed the essence of cooperatives by interpreting the basic concepts of cooperatives, analyzing the basic characteristics of cooperatives, and comparing cooperatives with companies and even questioned whether there is a real cooperative [7]. The third is the research on the governance structure of cooperatives. The research on the governance structure of cooperatives stems from the discussion of the normative issues of cooperatives. Scholars have discussed the problems of organizational alienation and organizational management in the development of cooperatives from the perspectives of stakeholders, corporate governance, and external environment. Scholars not only demonstrated the importance of factors such as product characteristics, members, production commercialization, and institutional environment for the establishment and development of farmers' professional cooperatives, but also attributed the main factors restricting the development of cooperatives to issues of legality, financing, and resources, with endowment problem [8]. The fourth is the research on the performance evaluation of cooperatives. The discussion of cooperative performance not only is an examination of the actual development of cooperatives, but also helps to provide top-level design guidance for the development of cooperatives. Scholars have not only constructed evaluation indicators, evaluation systems, and evaluation frameworks for the performance evaluation of cooperatives, but also demonstrated the impact of human capital, social capital, and other factors on the performance of cooperatives [9]. Internationally, it is obviously more in depth at the theoretical level of the research problem. In the early stage of cooperative development, Western scholars also focused on the mechanism and standardization of cooperatives [10]. However, with the gradual maturity of cooperatives, the research on cooperatives is no longer limited to the interpretation of their own rationality, and cooperatives have begun to be included as economic subjects into broader research categories, such as in economics, management, and organizational behavior. Cooperatives can be seen in fields such as applied technology [11]. This can be seen from keywords such as governance, collective action, management, and market in the visual analysis. And on some issues, there are big differences between Chinese and international cooperative research. For example, Western countries give cooperatives more corporate attributes, or cooperatives are enterprises in some countries. This can also be demonstrated from the keyword “firm” in hot research. For another example, Western scholars regard the governance structure of cooperatives as a part of the property rights structure, especially referring to the control rights of cooperatives [12].

From a theoretical point of view, the research on cooperatives has not yet jumped out of the previous theoretical category in the West. It is more of an explanation of the current situation and problems by borrowing original theories and has not formed a theoretical context based on its own developmental characteristics of cooperatives. It has obvious shortcomings in theoretical depth [13]. The result of this is that the theoretical research on cooperatives lacks innovation and a systematic theoretical research system [14]. When discussing the service function of cooperatives, scholars mostly consider the service effect or the final economic effect. Instead, they regard the service function of cooperatives as a common-sense judgment and categorize them into product categories. Before, during, and after childbirth, or the production service function and the operational service function, it ignores the discussion of more in-depth theoretical issues such as the nature and evolution mechanism of the cooperative service function [15]. This apparent cognition of the phenomenon makes the current research lack theoretical depth, and it is even more difficult to trace the causes and consequences of the dilemma in the evolution of cooperative service functions. The development of cooperatives is in the transition period of agricultural economy and has its own unique political, economic, and cultural background [16]. Therefore, the research on the evolution of cooperatives' service functions should first be combined with the background of the times, trace them from a deeper theoretical level, and clearly identify cooperatives causality in the evolution of service functions as a basis for practice [17]. As a kind of mutual aid economic organization with the attributes of a legal person but not for profit, it is too one-sided to interpret the service function of cooperatives from the perspective of market substitution or market complementarity. The conflict of goals between them creates a dilemma in understanding the positioning of cooperatives [18]. Literature [19] builds a systematic theoretical analysis framework for the evolution of cooperative service functions by summarizing and condensing existing relevant theories, in order to get rid of the current apparent cognition of cooperative service functions and explore economic phenomena and organizations from a deeper theoretical level behavior.

## 3. The Basic Working Principle and Main Performance Index of Intelligent Data Sampling ADC

**Figure 1:**
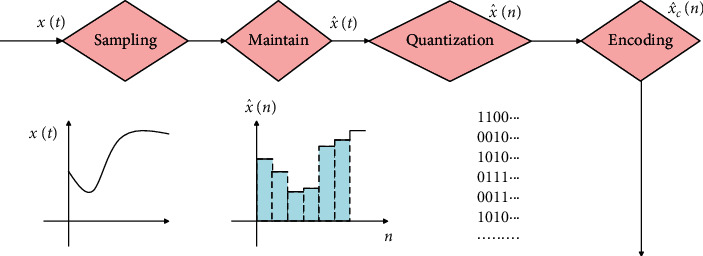
ADC working flowchart.

Analog-to-Digital Converter (ADC) is a device that converts analog signals into digital signals. The conversion process can be divided into four steps: sampling, holding, quantizing, and encoding. Sampling is the process of acquiring the amplitude of the input analog signal at a certain moment, that is, changing the analog signal into a series of narrow pulses. The amplitude of the pulse depends on the amplitude of the analog signal at the sampling moment. The signal after sampling is a series of sample pulses. The width of the sample pulse is generally very short. Before the next sample pulse arrives, the acquired sample pulse amplitude should be temporarily maintained for conversion. Therefore, a holding circuit must be added after the sampling circuit to get the ladder wave. At this time, the amplitude of the step wave is still the actual amplitude of the sampling point, so it is an analog quantity, and there are countless kinds of values. Due to the limited number of digital quantization bits, it can only represent a limited number of values (n-digit numbers can only represent 2*n* values at most), so the sample level after sampling must be normalized to a discrete level close to it. The process is called quantification. The quantized discrete value is represented by n-bit binary numbers. This process is called encoding. The analog-to-digital conversion process is shown in Figure 1.

The performance index of ADC can be divided into static characteristic index and dynamic characteristic index. Static indicators include resolution, accuracy, Offset Error, Gain Error, Differential Nonlinearity (DNL), and Integral Nonlinearity (INL). The dynamic characteristic index generally refers to its transmission parameter, which represents the digital reproduction capability of the analog input waveform of the device. It mainly includes signal-to-noise ratio (SNR), signal-to-noise and distortion ratio (SNDR), effective number of bits (ENOB), and spurious free dynamic range (SFDR). This section will introduce some characteristic indexes related to this paper.

### 3.1. Resolution and Accuracy

The resolution of the ADC refers to the smallest amount of change in the amplitude of the analog signal that the ADC can distinguish. It can be expressed as a percentage of the full scale. Usually we use the number of quantization bits *n* to indicate that the converter has 2n possible output states.

The accuracy of the ADC refers to the number of bits output by the ADC converter. For example, an ideal n-bit ADC converter has an accuracy of *n*.

### 3.2. Offset Error

**Figure 2:**
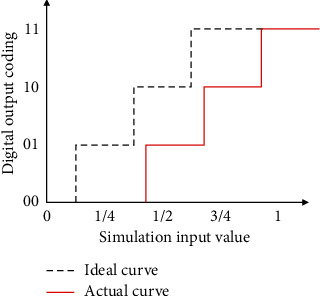
Schematic diagram of offset error.

In the actual ADC circuit, the input, output, and comparator of the operational amplifier have certain inherent offset currents and voltages. These offset currents and voltages are caused by the mismatch of the device. When the input is zero, these mismatches will cause the output to show nonzero values, as shown in Figure 2.

### 3.3. Gain Error

**Figure 3:**
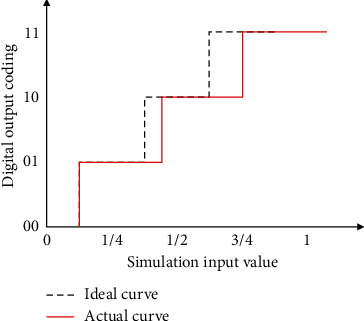
Schematic diagram of gain error.

The gain error refers to the difference between the actual characteristic curve and the theoretical characteristic curve under the assumption that no other errors exist, and it is proportional to the amplitude of the input voltage, as shown in Figure 3.

### 3.4. Signal-to-Noise Ratio

The signal-to-noise ratio is the ratio of the power of the signal to the noise. The signal-to-noise ratio of the ADC is defined as the ratio of the power of the signal component to the noise power in the spectrum. Here, noise does not include DC components and harmonic components. It is expressed as

(1) $$\begin{array}{c} \text{SNR}=10\;\log_{10}\left(\frac{P_{s}}{P_{n}}\right). \end{array}$$

### 3.5. Signal-to-Noise and Distortion Ratio

The signal-to-noise and distortion ratio (SNDR) represents the ratio of the signal power to the power of all the remaining noise and harmonics, expressed as

(2) $$\begin{array}{c} \text{SNDR}=10\;\log_{10}\left(\frac{P_{s}}{P_{n}+\Sigma_{k=1}^{\infty }P_{h}}\right). \end{array}$$

### 3.6. Effective Number of Bits

Effective number of bits represents the number of bits that the ADC outputs without errors and represents the actual accuracy of the ADC. Its calculation expression is

(3) $$\begin{array}{c} \text{ENOB}=\frac{\text{SNDR}-1.76}{6.02}. \end{array}$$

### 3.7. Spurious Free Dynamic Range

Spurious free dynamic range refers to the ratio of the amplitude of the fundamental wave component to the amplitude of the largest harmonic signal. When the system contains not only the fundamental wave component but also the frequency spectrum component with a small amplitude, if its amplitude is higher than the maximum harmonic, it can be detected; otherwise it cannot be detected. Therefore, the spurious free dynamic range represents the signal amplitude range that the ADC can recognize. The calculation expression is

(4) $$\begin{array}{c} \text{SFDR}=10\;\log_{10}\left(\frac{P_{s}}{\max\left(P_{h}\right)}\right). \end{array}$$

As the bandwidth of the intelligent data signal increases, the system has higher and higher requirements for the signal sampling rate. TI-ADC is an architecture that gets rid of the limitation of the sampling rate and resolution of a single ADC chip. It uses multiple low-speed ADC chips for time-sampling of the analog signal and then performs data splicing and recombination to achieve the purpose of high-speed sampling.

**Figure 4:**
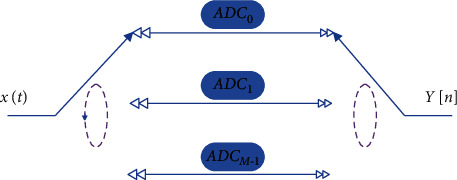
TI-ADC architecture diagram.

**Figure 5:**
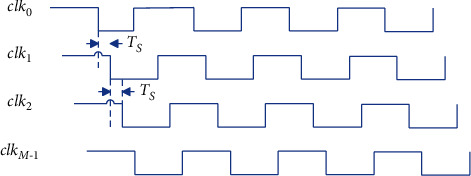
TI-ADC timing diagram.

The TI-ADC system consists of *M* ADC chips and input and output path gate switches. The strobe switch sequentially gates *M* ADC chips, the interval between two adjacent sampling clocks is *T*_*S*_, the input analog signal is *x*(*t*), and the final output high sampling rate digital signal is *y*[*n*] g. The system architecture and sampling timing diagram are shown in Figure 4 and Figure 5, respectively.

Since the sampling period of the system is *T*_*S*_, the sampling period of the single-chip ADC is ${\hat{T}}_{S}=M*T_{S}$. Therefore, the sampling time of the m-th sub-ADC can be obtained as

(5) $$\begin{array}{c} t_{m}\left[n\right]=n\hat{T_{S}}+mT_{S}=\left(nM+m\right)T_{S}. \end{array}$$

Therefore, the output of the m-th ADC is

(6) $$\begin{array}{c} \hat{y_{m}}\left[n\right]=x\left(t_{m}\left[n\right]\right)=x\left(\left(nM+m\right)T_{S}\right). \end{array}$$

The output signals of all the subchannels are added to obtain the final output signal; that is, the output ${\hat{y}}_{m}\left[n\right]$ of each sub-ADC composes *y*[*n*] together, which can be obtained:

(7) $$\begin{array}{c} y\left[n\right]=\hat{y_{m}}\left[\frac{n-m}{M}\right]\;m=n\mathrm{mod}M. \end{array}$$

We define the *M* multiplication sampling sequence of $\hat{y_{m}}\left[n\right]$ as *y*_*m*_[*n*], so that

(8) $$\begin{array}{c} y_{m}\left[n\right]=\left\{\begin{array}{cc} \hat{y_{m}}\left[\frac{n-m}{M}\right], & \frac{n-m}{M}\text{ Is an integer,}\\ 0, & \text{else}. \end{array}\right) \end{array}$$

For convenience, we define the sampling sequence as

(9) $$\begin{array}{c} \delta_{m}\left[n\right]=\sum_{k=-\infty }^{\infty }\delta \left[n-kM-m\right]. \end{array}$$

Therefore,

(10) $$\begin{array}{c} y_{m}\left[n\right]=x\left(nT_{S}\right)\delta_{m}\left[n\right]. \end{array}$$

Therefore, the output *y*[*n*] of TI-ADC can be written as

(11) $$\begin{array}{c} y\left[n\right]=\sum_{m=0}^{M-1}y_{m}\left[n\right]. \end{array}$$

It can be seen from (11) that the output of an ideal TI-ADC is *y*[*n*]=*x*(*nT*_*S*_).

Above, we analyze and deduce the theoretical feasibility of TI-ADC architecture from the perspective of the time domain. The following will further verify our point of view by analyzing the characteristics of the signal in the frequency domain.

Discrete-time Fourier Transform (DTFT) is often used to represent the TI-ADC discrete-time output *y*[*n*] in the frequency domain and the output *y*_*m*_[*n*] of the sub-ADC. Generally speaking, the DTFT of *x*[*n*] can be expressed as

(12) $$\begin{array}{c} X\left(e^{j\omega }\right)=\sum_{n=-\infty }^{\infty }x\left[n\right]e^{-j\omega n}. \end{array}$$

Among them, *X*(*e*^*jω*^) is a continuous function with period 2*π*.

The output sequence *y*_*m*_[*n*] of the m-th ADC is subjected to DTFT conversion, and then

(13) $$\begin{array}{c} Y_{m}\left(e^{j\omega }\right)=\sum_{n=-\infty }^{\infty }y_{m}\left[n\right]e^{-j\omega n}. \end{array}$$

By substituting formula (10), we can get

(14) $$\begin{array}{c} Y_{m}\left(e^{j\omega }\right)=\sum_{n=-\infty }^{\infty }\left(x\left(n\right)\delta_{m}\left[n\right]\right)e^{-j\omega n}. \end{array}$$

Among them, *x*[*n*]=*x*(*nT*_*S*_). It can be seen from the nature of DTFT that *Y*_*m*_(*e*^*jω*^) is equivalent to the convolution of the DTFT of *x*[*n*] and *δ*_*m*_[*n*]. If the DTFT of *x*[*n*] is assumed to be *X*(*e*^*jω*^), the DTFT of *δ*_*m*_[*n*] is

(15) $$\begin{array}{c} D_{m}\left(e^{j\omega }\right)=\frac{2\pi }{M}\sum_{k=-\infty }^{\infty }\delta \left(\omega -\frac{2\pi k}{M}\right)e^{j\left(2\pi k/M\right)m}. \end{array}$$

Therefore, we can get

(16) $$\begin{array}{c} Y_{m}\left(e^{j\omega }\right)=\frac{1}{2\pi }\int_{-\pi }^{\pi }X\left(e^{j\theta }\right)D_{m}\left(e^{j\left(\omega -\theta \right)}\right)\text{d}\theta =\frac{1}{M}\overset{\infty }{\underset{k=-\infty }{\sum }}e^{j\left(2\pi k/M\right)m}X_{\text{main }}\left(e^{j\left(\omega -2\pi k/M\right)}\right). \end{array}$$

Among them, *X*_main_(*e*^*jω*^) is the function of *X*(*e*^*jω*^) in the main interval [−*π*, *π*]. From this result, it can be seen that *Y*_*m*_(*e*^*jω*^) is obtained by shifting the main value part of *X*(*e*^*jω*^) with 2*πk*/*M* as the period. It can be further seen that each sub-ADC has the same amplitude-frequency response, but the phase-frequency response function of each channel is different. The DTFT of TI-ADC output *y*[*n*] in formula (7) can be expressed as

(17) $$\begin{array}{c} Y\left(e^{j\omega }\right)=\sum_{m=0}^{M-1}Y_{m}\left(e^{j\omega }\right). \end{array}$$

By substituting formula (16), we can get

(18) $$\begin{array}{c} Y\left(e^{j\omega }\right)=\overset{\infty }{\underset{k=-\infty }{\sum }}M\left[k\right]X_{\text{main }}\left(e^{j\left(\omega -2\pi k/M\right)}\right),\\ M\left[k\right]=\frac{1}{M}\overset{M-1}{\underset{m=0}{\sum }}e^{j\left(2\pi k/M\right)m}=\left\{\begin{array}{cc} 1, & \frac{k}{M}\text{Is an integer,}\\ 0, & \text{else}. \end{array}\right) \end{array}$$

Therefore,

(19) $$\begin{array}{c} Y\left(e^{j\omega }\right)=\sum_{k=-\infty }^{\infty }X_{\mathrm{main}}\left(e^{j\left(\omega -2\pi k\right)}\right)=X\left(e^{j\omega }\right). \end{array}$$

It can be seen from the above formula that the final combined output *Y*(*e*^*jω*^) is the translation of the DTFT main value interval of *x*[*n*], that is, the DTFT of *x*[*n*]. This also proved the theoretical feasibility of TI-ADC.

Above, we have verified the theoretical feasibility of TI-ADC architecture from the perspective of time domain and frequency domain. Below, we will model the TI-ADC architecture for error analysis and correction. In general, the TI-ADC model can be divided into a time-varying filter (TVF) model and a hybrid filter bank (HFB) model using multirate signal processing theory. Since the HFB model can easily derive the signal input and output spectrum relationship, which is beneficial to the analysis and correction of the mismatch error, this paper chooses the HFB model to model the TI-ADC system.

**Figure 6:**
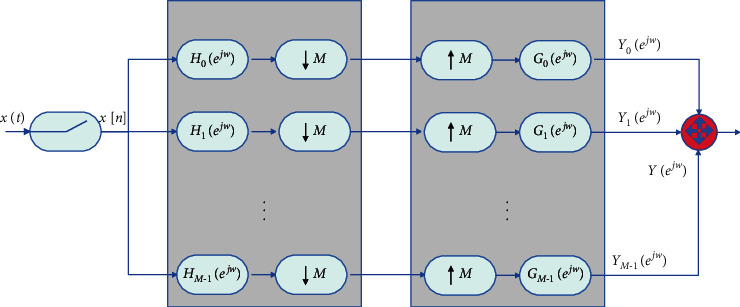
HFB model.

The HFB model corresponding to TI-ADC is shown in Figure 6. The multirate filter bank is obtained on the basis of decimation and interpolation. The filter components are decomposition filter bank (FB) and integrated filter bank (SFB). The decomposition filter bank includes a module with a response function of *H*_*m*_(*e*^*jωT*_*s*_^) and a decimator, and the comprehensive filter bank includes an internal decimator and a module with a response function of *G*_*m*_(*e*^*jωT*_*s*_^).

If it is assumed that the input and output of the decimator in the decomposition filter bank AFB are *u*[*n*] and *v*[*n*]; the relationship between the input and output is

(20) $$\begin{array}{c} V\left(e^{j\omega }\right)=\frac{1}{M}\sum_{k=0}^{M-1}U\left(e^{j\left(\omega /M-2\pi k/M\right)}\right). \end{array}$$

Thus, it can be obtained that the DTFT of the output signal after the m-th channel *x*[*n*] passes through the AFB is

(21) $$\begin{array}{c} X_{m}\left(e^{j\omega T_{s}}\right)=\frac{1}{M}\sum_{k=0}^{M-1}X\left(e^{j\left(\omega T_{s}/M-2\pi k/M\right)}\right)H_{m}\left(e^{j\left(\omega T_{s}/M-2\pi k/M\right)}\right). \end{array}$$

Similarly, if it is assumed that the input and output of the upsampling internal regulator of the integrated filter bank are *u*[*n*] and *v*[*n*], the relationship is

(22) $$\begin{array}{c} V\left(e^{j\omega }\right)=U\left(e^{j\omega L}\right). \end{array}$$

Therefore, the output of the signal after passing through the SFB is

(23) $$\begin{array}{c} Y_{m}\left(e^{j\omega T_{s}}\right)=X_{m}\left(e^{j\omega MT_{s}}\right)G_{m}\left(e^{j\omega T_{s}}\right). \end{array}$$

In summary, the output of TI-ADC is

(24) $$\begin{array}{c} Y\left(e^{j\omega T_{s}}\right)=\sum_{m=0}^{M-1}Y_{m}\left(e^{j\omega T_{s}}\right),\\ =\frac{1}{M}\sum_{m=0}^{M-1}\sum_{k=0}^{M-1}X\left(e^{j\left(\omega T_{s}-2\pi k/M\right)}\right)H_{m}\left(e^{j\left(\omega T_{s}-2\pi k/M\right)}\right)G_{m}\left(e^{j\omega T_{s}}\right). \end{array}$$

When the system reaches a perfect matching state, there are the following equations:

(25) $$\begin{array}{c} H_{m}\left(e^{j\omega T_{s}}\right)=e^{j\omega mT_{s}},\\ G_{m}\left(e^{j\omega T_{s}}\right)=e^{-j\omega mT_{s}}. \end{array}$$

By substituting it into formula (24), we can get

(26) $$\begin{array}{c} Y\left(e^{j\omega T_{s}}\right)=\sum_{k=0}^{M-1}X\left(e^{j\left(\omega T_{s}-2\pi k/M\right)}\right)\frac{1}{M}\sum_{m=0}^{M-1}e^{-j2\pi km/M}. \end{array}$$

This is because

(27) $$\begin{array}{c} \frac{1}{M}\sum_{m=0}^{M-1}e^{-j2\pi km/M}=\left\{\begin{array}{cc} 1, & k=0,\pm M,\pm 2M\ldots ,\\ 0, & k\neq 0. \end{array}\right) \end{array}$$

Thus, we can get

(28) $$\begin{array}{c} Y\left(e^{j\omega T_{s}}\right)=X\left(e^{j\omega T_{s}}\right). \end{array}$$

That is, when the system reaches a perfect reconstruction state, the output of TI-ADC is equivalent to a sequence obtained by sampling a single ADC with TS as the sampling period.

## 4. Ecosphere Management Model of Farmer Cooperatives Based on Intelligent Data Sampling Technology

**Figure 7:**
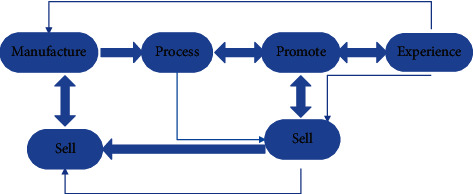
Flowchart of the ecosphere model of farmer cooperatives.

During the operation of the ecological circle, in the ecological operation of nature, there are interrelationships between the ecological animals and plants at each layer and other animals and plants. This cross-correlation relationship maintains the benign operation of the ecological circle. The overall plan of the ecosystem, in terms of specific projects, is the comprehensive operation of multifunctional and multiformat business structures. It goes beyond the original single-line thinking to cross-industry and multifunctional. For example, it used to be a piece of farmland, but now it needs to have tourism momentum, and it used to be a house, but now it is possible to open an inn at the same time. The ecosphere breaks through the original habitual use and must break through habitual thinking in ideology and conform to cultural development, technological progress, market evolution, and institutional innovation. It is to combine the exploration of supply-side reforms to stimulate the effectiveness of originally limited assets and resources and form a broad space for the development of rural social industries. The assets mentioned here include rural land, houses, and cultural assets. The following improvements have been made to the ecological operation of cooperatives, as shown in Figure 7.

**Figure 8:**
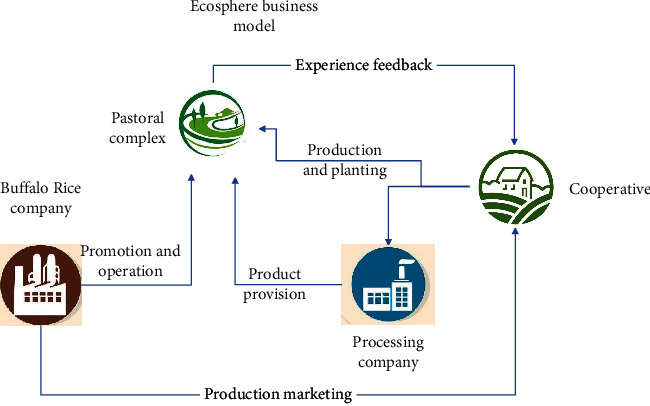
Ecosystem business model diagram of farmer cooperatives.

Figure 8 shows that the operating model of the ecosystem must focus on the pastoral complex. Whether it is cooperative planting, processing plant processing, or marketing and promotion of operating companies, the core convergence point is the pastoral complex. One is to provide support for other industries by guiding processing and production and to provide space for cooperative technological research, processing, and production. The second is to promote brand product promotion experience through the cultural and tourism industry. The third is to improve service experience and obtain operating income.

**Figure 9:**
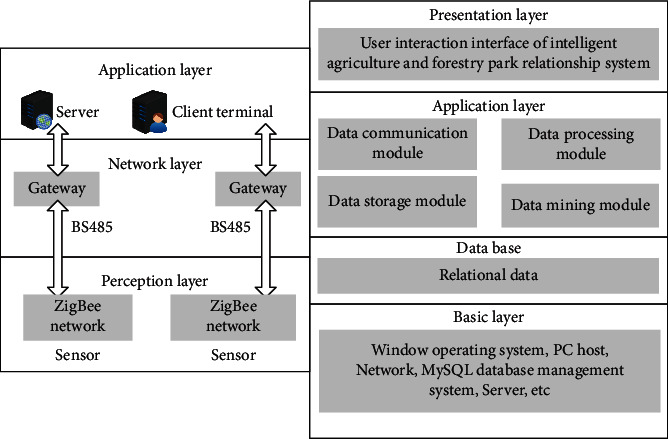
Intelligent data sampling platform architecture.

As shown in Figure 9, the overall architecture of the platform consists of four major parts, namely, the base layer, the database layer, the application layer, and the display layer. What the basic layer builds is the basic information layer of the basic system. The hardware of this layer is composed of three parts: server, PC, and network facilities. The corresponding software is to configure the corresponding operating system and data inventory management system for these two parts. The network protocol forms the network facility into a communication network entity. In the database layer, this design adopts relational database as the basic form of database construction. The reason is that relational database is a mature technology and highly reliable database construction form. For most developers, the familiarity is very high, which is convenient for system expansion and maintenance. In addition, the application layer is the key layer that forms the system application, which mainly completes data communication, processing, storage, and mining. This part is the center bar between the bottom and upper layers of the system. In the design, the main function of the application layer is set on the server. Finally, at the display layer, the display layer of the system is used to form a variety of display methods. In this design, the PC display method is adopted, and Java is used as the development language. Of course, the corresponding App program can be developed as another display method according to the needs in the later period.

**Figure 10:**
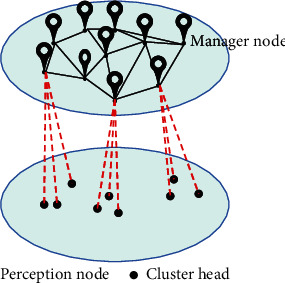
System hierarchical network topology.

A single-layer network will inevitably lead to an increase in system cost and a waste of performance. However, the profitability of agricultural production is meager and very sensitive to equipment costs. Moreover, the excessively high cost will cause the system to be unsuitable for application in the production of smart agriculture. Therefore, this system adopts a two-layer network topology, the upper layer is the cluster head node, and the lower layer is the sensor node. The upper layer uses a self-organizing Zigbee network structure to complete the communication between the sensor nodes in the cluster, and at the same time, it can be connected to the external network through the Zigbee network to transmit data to the management node. The lower layer adopts a star network structure and uses flooding routing protocol to complete the communication between the cluster head node and the sensor nodes in the cluster. Moreover, it introduces the concept of link correlation to improve the traditional flooding protocol, reduces the power consumption of the flooding protocol, improves the efficiency, and solves the ACK implosion problem. The experimental results prove that the network topology is very suitable for use and promotion in smart agricultural production. The hierarchical network topology of this system is shown in Figure 10.

**Figure 11:**
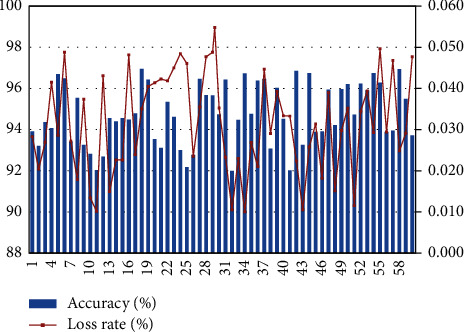
Statistical diagram of the effect of smart data sampling technology.

After constructing the above system model, the ecosphere management model of farmer cooperatives based on intelligent data sampling technology is studied. Moreover, this paper conducts simulation research on smart data adoption technology, counts the accuracy of smart data sampling, and calculates the data loss rate, and obtains the results shown in Table 1 and Figure 11.

**Figure 12:**
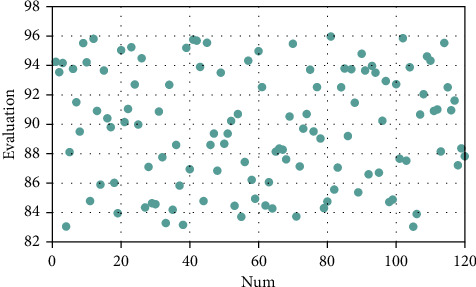
Evaluation of the effect of the ecosphere management model of farmer cooperatives based on intelligent data sampling technology.

As shown in the figure above, the bar graph represents the sampling accuracy of smart data (primary axis), the line graph represents the loss rate of smart data sampling (secondary axis), and the abscissa is the experimental group number. It can be seen from the above experiments that, on the basis of the above research, this paper evaluates the effect of this system in the ecosphere management model of farmer cooperatives and conducts data processing in the way of expert evaluation and obtains the results shown in Figure 12.

From the above analysis, it can be seen that the ecosphere business model of farmer cooperatives based on intelligent data sampling technology proposed in this paper has good results.

## 5. Conclusion

The growth and development process of farmer cooperatives is deeply embedded in the institutional environment. Taking farmer cooperatives as the research object can not only gain insight into the ecological circle and operating mechanism constructed by the internal subjects of farmer cooperatives, but also observe the niche forms constructed by external subjects such as farmer cooperatives and institutional actors and agricultural supply chain subjects. Based on this, this paper attempts to construct a new theoretical framework based on organizational ecology, etc., to analyze and explain the establishment and evolution of farmer cooperatives. Moreover, this paper combines the intelligent data sampling technology to analyze the ecosphere operation mode of farmers' cooperatives, constructs an intelligent system, and analyzes the further development of the ecological circle operation mode of farmers' cooperatives. The experiment results show that the ecosphere business model of farmer cooperatives based on intelligent data sampling technology proposed in this paper has good results.

## Figures and Tables

**Table 1:** Statistical table of performance parameters of intelligent data sampling technology.

Num	Accuracy (%)	Loss rate (%)	Num	Accuracy (%)	Loss rate (%)
1	93.918	0.028	31	96.426	0.023
2	93.209	0.020	32	92.002	0.011
3	94.367	0.027	33	94.476	0.023
4	94.071	0.042	34	96.737	0.010
5	96.700	0.029	35	94.764	0.027
6	96.498	0.049	36	96.391	0.021
7	93.418	0.027	37	96.477	0.045
8	95.547	0.018	38	93.073	0.029
9	93.264	0.037	39	96.036	0.039
10	92.826	0.013	40	94.522	0.033
11	92.030	0.010	41	92.012	0.033
12	92.692	0.043	42	96.855	0.022
13	94.564	0.015	43	93.256	0.011
14	94.404	0.023	44	96.750	0.026
15	94.560	0.023	45	93.886	0.031
16	94.491	0.048	46	93.919	0.018
17	94.790	0.024	47	95.946	0.039
18	96.965	0.035	48	94.223	0.015
19	96.433	0.041	49	95.973	0.030
20	93.533	0.041	50	96.216	0.035
21	93.121	0.042	51	94.729	0.012
22	95.347	0.042	52	96.240	0.034
23	94.625	0.045	53	95.915	0.039
24	93.008	0.048	54	96.757	0.029
25	92.183	0.046	55	96.286	0.050
26	92.771	0.024	56	93.889	0.029
27	96.471	0.035	57	93.951	0.047
28	95.681	0.048	58	96.940	0.025
29	95.663	0.049	59	95.504	0.029
30	94.736	0.035	60	93.727	0.048

## Data Availability

The labeled datasets used to support the findings of this study are available from the corresponding author upon request.
